# Action Categorization in Rhesus Monkeys: discrimination of grasping from non-grasping manual motor acts

**DOI:** 10.1038/s41598-017-15378-6

**Published:** 2017-11-08

**Authors:** Koen Nelissen, Wim Vanduffel

**Affiliations:** 1 Laboratory for Neuro- & Psychophysiology, Department of Neurosciences, KU Leuven, Leuven, 3000 Belgium; 2 Massachusetts General Hospital, Harvard Medical School, Athinoula A. Martino’s Center for Biomedical Imaging, Charlestown, Massachusetts, 02129 USA

## Abstract

The ability to recognize others’ actions is an important aspect of social behavior. While neurophysiological and behavioral research in monkeys has offered a better understanding of how the primate brain processes this type of information, further insight with respect to the neural correlates of action recognition requires tasks that allow recording of brain activity or perturbing brain regions while monkeys simultaneously make behavioral judgements about certain aspects of observed actions. Here we investigated whether rhesus monkeys could actively discriminate videos showing grasping or non-grasping manual motor acts in a two-alternative categorization task. After monkeys became proficient in this task, we tested their ability to generalize to a number of untrained, novel videos depicting grasps or other manual motor acts. Monkeys generalized to a wide range of novel human or conspecific grasping and non-grasping motor acts. They failed, however, for videos showing unfamiliar actions such as a non-biological effector performing a grasp, or a human hand touching an object with the back of the hand. This study shows the feasibility of training monkeys to perform active judgements about certain aspects of observed actions, instrumental for causal investigations into the neural correlates of action recognition.

## Introduction

The ability to recognize other individuals’ actions is of significant importance for primates and a fundamental aspect of social behavior. Rhesus monkeys’ ability to recognize, or even comprehend actions in terms of goals and intentions, is often assumed in many neurophysiological studies that use action observation as a proxy for action recognition or comprehension^1–7^. Since most of these studies did not include a behavioral response, it is difficult to quantify whether monkeys actually recognize these observed motor acts and to what degree neural activity during these observation tasks reflects action understanding. Several protocols have been used to study the monkey’s action comprehension abilities in a more ethological setting. These include experiments during which monkeys observed accidental versus intentional actions, rational versus irrational actions or actions performed by actors in an unwilling versus an incapable situation. Monkey behavioral responses measured during these experiments include different paradigms such as looking-time, forced-choice food foraging, or observation of monkey’s vocal and body responses towards the actor^8–13^. Most of these behavioral studies, however, were performed in a setting not easily compatible with simultaneous recording of brain activity.

While several action recognition models have been proposed (for recent review, see^14^) to date the exact mechanisms and detailed computations performed by the brain underlying action recognition, are far from understood. To gain further insights into the neuronal correlates of action recognition, tasks will be required that allow measuring functional brain responses not only when subjects passively observe actions, but when they have to make behavioral judgements related to the observed actions^15–18^. Combining these behavioral tasks with reversible perturbations^19^ will provide much needed causal evidence of specific visuo-motor nodes roles in action recognition. For instance, numerous studies using categorization tasks combined with focal reversible perturbations have allowed examining the causal role of certain brain regions in the perception of specific stimulus properties or categories in monkeys^20–23^. In humans on the other hand, in particular TMS in combination with behavioral tasks has been employed to provide causal evidence for specific visuo-motor contributions to different aspects of action recognition^24–26^, for review see^27^.

**Figure 1:**
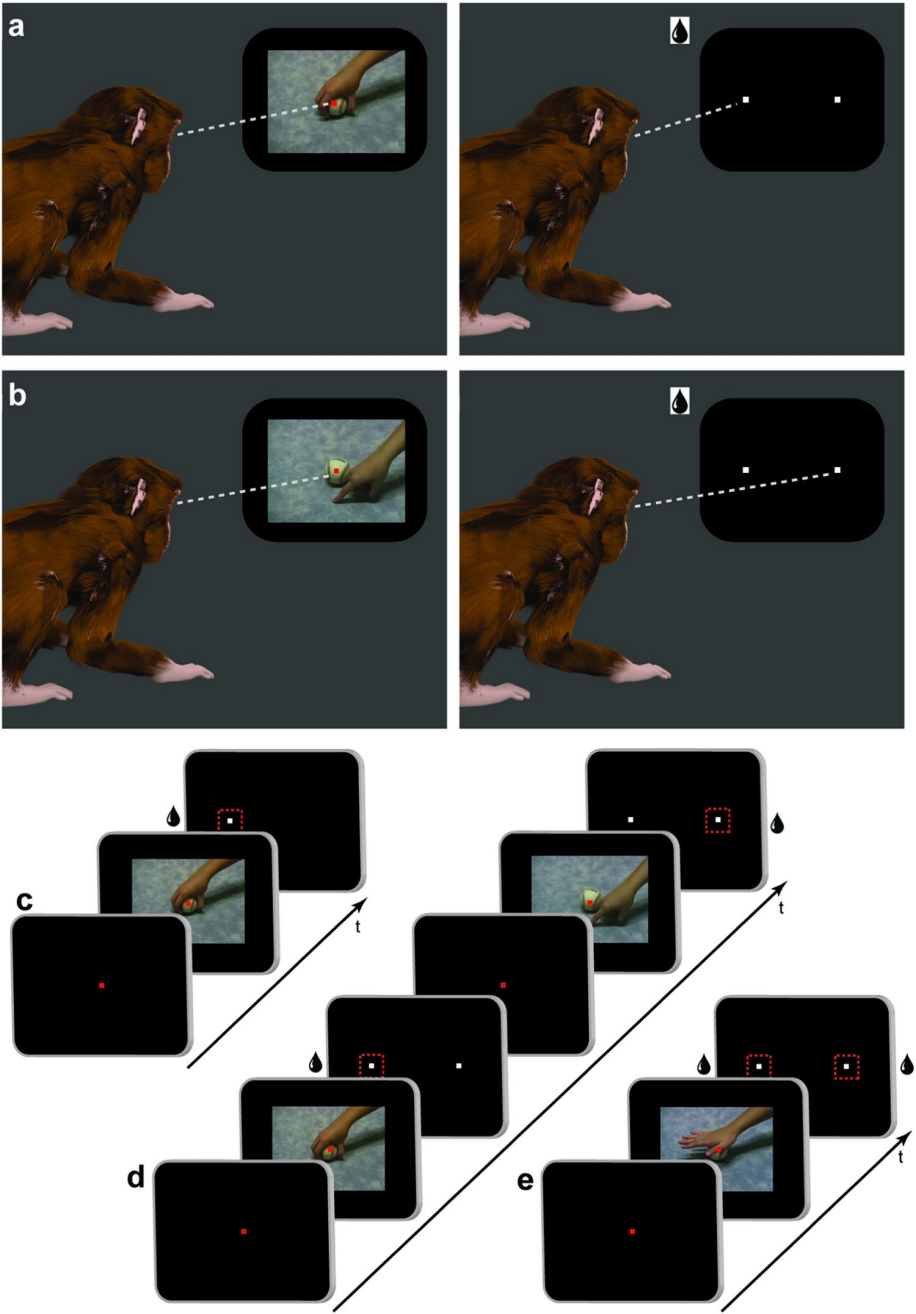
Two-alternative categorization task training and generalization testing procedure. Monkeys were required to fixate a video on a screen positioned in front of them showing either a grasping (**a**) or a non-grasping (**b**) manual motor act. After video presentation, the video disappeared and two peripheral targets appeared on the screen. A liquid reward was given if monkeys made a saccade to the correct target (left for grasping, right for non-grasping motor acts). Monkey renderings in (**a**) and (**b**) were made using open-source software Blender (https://www.blender.org/). (**c**) In the first training sessions, during a proportion of categorization trials (up to 30%) only the correct target was shown after video presentation (for illustration only a grasping trial is shown). A saccade to this target would be rewarded. The remainder of the trials consisted of two targets as shown in (**d**). The red dashed squares around the targets in A,B and C were not physically shown and are for illustration purpose only to indicate a saccade to that target would result in a reward. (**d**) During later training sessions, after video presentation, two targets were shown simultaneously and a reward was given for a saccade to the correct target. (**e**) During generalization testing, after video presentation, two targets would appear and selection of either target would be rewarded in order to avoid the monkey learning the novel stimuli.

In this behavioral study, we investigated rhesus monkeys’ ability to discriminate different types of motor acts. In a two-alternative forced-choice action categorization task, monkeys learned to discriminate grasping motor acts (Fig. 1a, Supplementary Video S1) from other manual non-grasping motor acts including touch with a finger (Fig. 1b, Supplementary Videos S2 and S5), touch with a closed fist (Supplementary Video S3), mimicked grasp next to an object (Supplementary Video S4). The monkeys indicated their choices by making a saccade to the left or right (Fig. 1a,b, right panels). After monkeys reached proficiency on this task (above 80% correct trials), we tested how well monkeys generalized this learned categorization rule (grasping versus non-grasping) to new, untrained videos of either grasping or non-grasping manual motor acts. During these generalization sessions, we tested if monkeys could discriminate untrained videos depicting 1) novel viewpoints of grasping and non-grasping motor acts, grasping with 2) novel objects and 3) novel effectors (human, conspecific or artificial), and non-grasping motor acts with 4) novel spatial positions of familiar hand configurations and 5) novel hand configurations.

## Methods

### Subjects

Two male (M1, M2) rhesus monkeys (Macaca mulatta, 4–6 kg, 3–5 years old) participated in the experiments. All animal care and experimental procedures met the national and European guidelines and were approved by the animal ethical committee of the KU Leuven.

### General Fixation Training

The monkey subjects were accustomed to sitting in a sphinx position in a plastic monkey chair, directly facing a liquid crystal display (LCD) screen (60 Hz frame rate), which was positioned at 57 cm from the monkeys‘ eyes^28,29^. During initial training, they were required to maintain fixation within a 2° × 2° window centered on a red fixation target (size: 0.18° × 0.18°) in the middle of the screen. Eye position was monitored at 120 Hz through pupil position and corneal reflection (Iscan). During this initial training phase, the monkeys were rewarded (fruit juice) for fixating the small red target within the fixation window for long periods (up to several minutes).

### Categorization task and training

The monkeys were trained to perform a two-alternative action categorization task, during which they had to discriminate videos of grasping motor acts from videos showing other manual non-grasping motor acts. Each categorization trial consisted of the following sequence of events: at the beginning of a trial, the monkey was required to fixate a small red fixation target (size: 0.18° × 0.18°) in the center of the screen, after which a video was presented with the fixation target superimposed (Fig. 1a,b, left panels). During video presentation, the monkey had to hold fixation within a 2° × 2° degree window centered on the fixation target. After presentation of the video, the central fixation target and the video were replaced by two peripheral white fixation targets (size: 0.18° × 0.18°), located along the horizontal meridian at 9.25° to the left or the right of the center of the screen. The monkey then had to make a saccade to one of the two targets in order to receive a juice reward (Fig. 1a,b, right panels). Grasping motor acts (Fig. 1a, Supplementary Video S1) were associated with the left target and non-grasping motor acts (Fig. 1b, Supplementary Videos S2–5) with the right target. Trials would be aborted if the monkey did not hold his gaze within the 2° × 2° central window for the entire video presentation or if monkey failed to saccade to one of the two peripheral targets within 2 seconds after stimulus presentations. These aborts were not incorporated into the data analysis.

**Figure 2:**
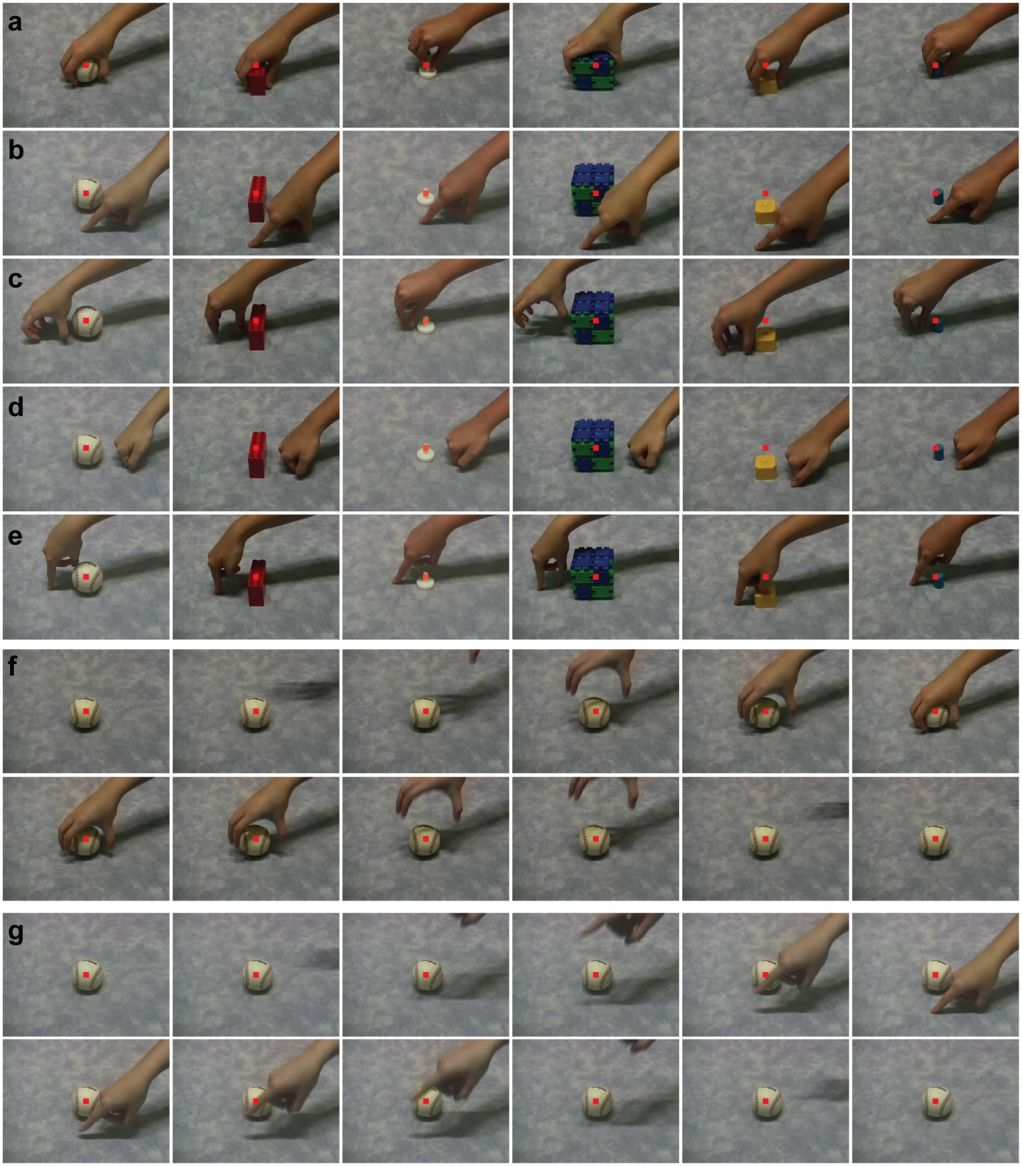
Visual stimuli used in the categorization task. (**a**) Six examples of different objects being *grasped*. (**b**–**e**) Six examples each of the different non-grasping motor acts: finger touch front (**b**), mimicked grasp (**c**), fist touch (**d**), finger touch side (**e**). (**f**) Frames of a grasping motor act: grasping videos showed a human hand approaching from the right and grasping a centrally-positioned object, after which the hand released the object and moved out of the frame. Monkeys were required to fixate the central small red spot superimposed on the video. (**g**) Frames of a non-grasping motor act: the non-grasping videos showed a human hand approaching from the right and touching the table without interacting with the object. In this example of a finger touch motor act, a finger touched the surface in front of the object, after which the hand moved out of sight.

During the first training sessions, in some of the trials (up to 30% of the trials) only the correct peripheral target was presented after the video (Fig. 1c). This way monkeys learned the general procedure of initial fixation followed by a saccade to a peripheral target. Through association, monkeys learned the correct target location for the grasping versus the non-grasping videos. Initial training started with only 2 videos, depicting grasping of a baseball (Fig. 1a, left panel) or a finger touching the surface in front of the baseball (Fig. 1b, left panel). Gradually, during the following training sessions, more stimuli were added to the stimulus set, depicting grasping motor acts with the 18 different objects (6 examples shown in Fig. 2a) or non-grasping motor acts consisting of the finger touch front, finger touch side, fist touch or mimicked grasps (6 examples shown in Fig. 2b–e). During training sessions, stimuli were picked randomly from this stimulus set. During these first categorization sessions, the rest of the trials consisted of two peripheral targets, and monkeys were rewarded for choosing the correct target. During later sessions, all trials consisted of two peripheral targets (Fig. 1d). In addition, we used a response-bias-correction procedure, similar to^15^. During this procedure, a trial in which an error occurred was followed by a trial of the same stimulus category. This procedure was maintained until the monkey made the correct response. Both monkeys typically performed around 600–700 trials during a daily training session.

### Stimuli

A total of 90 videos (grasping and non-grasping) were used in the training session. Grasping videos consisted of a human hand grasping diverse objects (18 different objects in total) with various grips including precision grip, 3-finger grasp, and whole hand grasp. Figure 2a shows example frames of 6 of the objects that were used. The non-grasping motor acts consisted of the same human hand touching the surface next to the same 18 objects used in the grasping videos. These non-grasping motor acts included touching the surface (table) with a finger (Fig. 2b,e), mimicking a grasp next to the object (Fig. 2c) or touching the surface next to the object with a closed fist (Fig. 2d). The grasping videos all started with a static, centrally-positioned object, followed by a human hand appearing in view and grasping the object, after which the hand released the object and disappeared out of view (Fig. 2f). A non-grasping video followed the same sequence of events: a video started with a static object, after which a hand came into view and touched the surface next to the object, and finally disappeared out of view (Fig. 2g, finger touch front). The videos measured 13.9 by 10.4 degrees, with a duration of 3 seconds.

### Generalization testing

After both monkeys had reached proficiency on the categorization task (consistently above 80% correct trials), we tested their abilities to generalize the learned categorization rule to novel, untrained examples of grasping and non-grasping manual motor acts. In 13 different generalization sessions, we tested if monkeys could correctly categorize untrained videos with 1) novel viewpoints, 2) novel objects, 3) novel effectors (human, conspecific or artificial), 4) novel spatial positions of familiar hand configurations and 5) novel hand configurations. Six generalization tests involved novel, untrained examples of grasping motor acts, while in the 7 additional tests, behavioral responses were tested towards novel, untrained non-grasping motor acts. During these generalization tests, around 90% of the trials consisted of trained videos (familiar) which were rewarded only on the correct side. The other 10% of the trials consisted of the novel untrained videos and were rewarded on both sides (Fig. 1e), to avoid the monkey also learning these novel stimuli^15,30^. No bias correction was used during these generalization sessions. During these generalization sessions, monkeys averaged 650 trials per session (which thus included ~65 trials with novel videos). For generalization tests 1 through 5, only one example of a novel motor act was used. For tests 6 through 13, the novel videos for that particular motor act consisted of the 18 different objects on which the monkeys had been previously trained (see methods).

## Results

### Categorization learning

**Figure 3:**
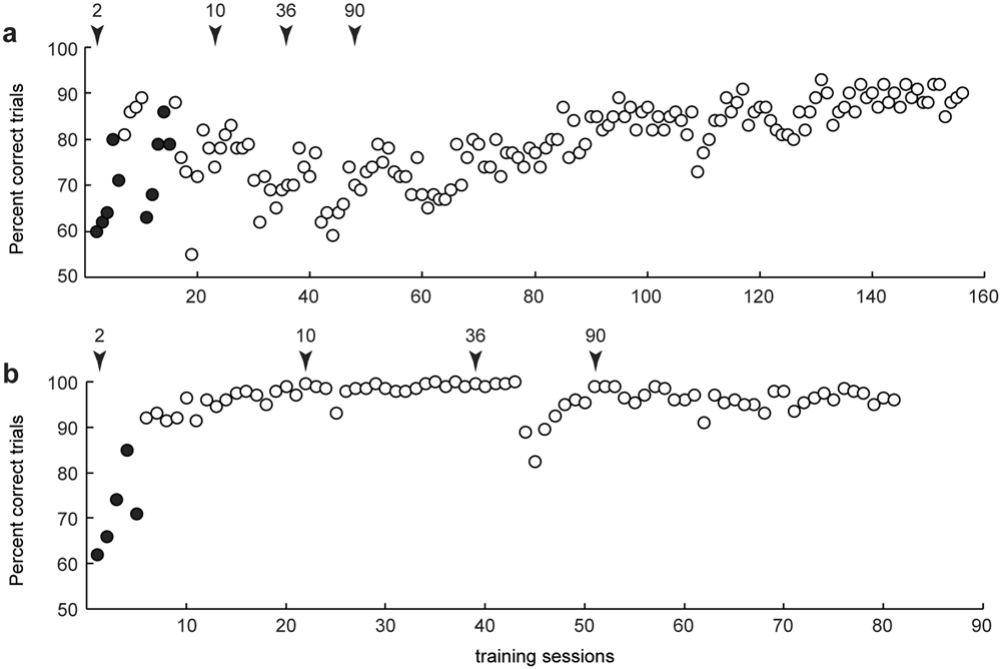
Performance as function of training session. Categorization training results of monkey M1 (**a**) and M2 (**b**). Percent correct trials are shown for different daily training sessions. Filled circles indicate session during which a proportion of trials (up to 30%) consisted of single targets (Fig. 1c). The open circles indicate sessions in which all trials consisted of two targets (Fig. 1d). A bias correction was used during training sessions only (see methods). Black arrows indicate total number of different videos in the training stimulus set.

Figure 3 shows the training curves of both monkeys. Filled circles indicate sessions during which a proportion of trials consisted of only a single target used to shape the animal’s response (see methods). Open circles indicate sessions during which all trials included both targets, in combination with a bias-correction (see methods). Initial training started with only two different videos (1 grasping, 1 non-grasping motor act) while in later phases more videos were gradually introduced. The arrows and corresponding numbers above the graphs (Fig. 3a,b) indicates the number of videos used during those time points in the course of the categorization training. While training in monkey M1 took several months (~90 sessions) before reaching a stable performance level using the 90 different videos (>80% accuracy), monkey M2 reached the same level of performance much sooner (i.e. after ~50 sessions).

### Generalization tests

**Figure 4:**
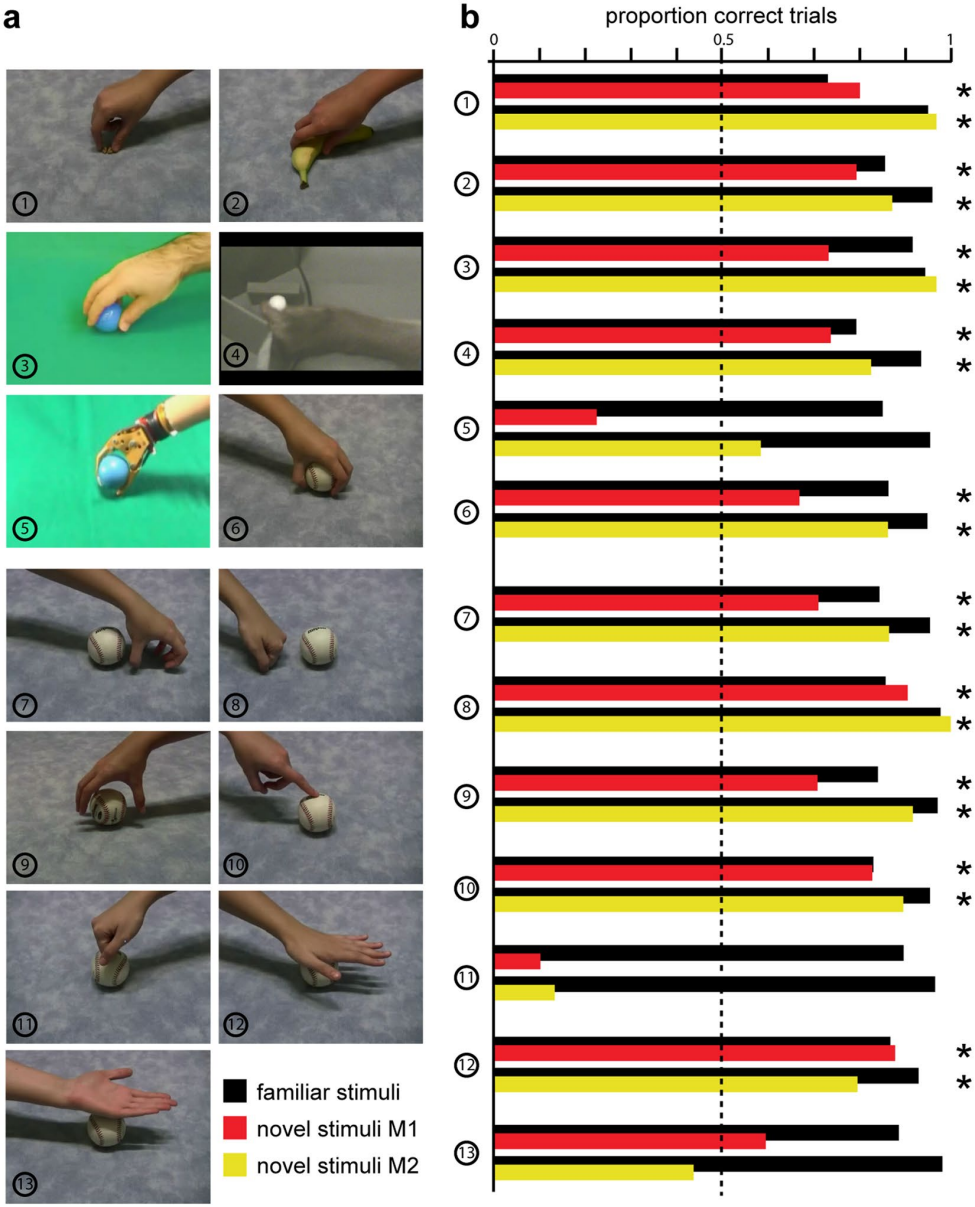
Generalization results. (**a**) Frames of the novel videos used during the 13 different generalization test sessions. In 6 different sessions, novel untrained videos showing a grasping motor act were used. These included an object being grasped with a precision grip (1) or a whole-hand grasp (2), a male actor grasping a ball (3), a monkey grasping a small cylinder (4), a prosthetic arm grasping a ball (5) and finally, mirrored versions of the familiar grasping videos (6). In 7 additional generalization tests, we tested generalization to novel, untrained non-grasping motor acts. These included mirror versions of the familiar mimicked grasp (7) and fist touch (8) videos, a hand initiating a grasp which halted immediately prior touching the object (9), a finger touching the object (10), a fist touching the object (11), an open hand touching the object with the palm (12) and an open hand touching the object with the back of the hand (13). **(b)** Proportion correct trials during the generalization tests. Black bars indicate performance of the monkeys during the 13 generalization tests for the trained familiar videos (90% of trials). Red (monkey M1) and yellow (monkey M2) bars indicate performance from the monkeys on the novel, untrained videos (10% of trials). Asterisks indicate significant generalization (p < 0.05, binomial test). No bias correction was used during the generalization sessions.

Next, we investigated whether monkeys could generalize to untrained examples of grasping and non-grasping manual motor acts. These generalization tests allowed us to examine whether the monkeys had merely memorized the familiar videos seen during the extensive training and to ascertain how specific the acquired categorization performance was for certain aspects of the videos. The results of the generalization tests are shown in Fig. 4. For generalization tests 1 through 5, only one novel video was used (frame shown in Fig. 4a). For tests 6 through 13, the novel videos for that particular motor act consisted of the 18 different objects on which the monkeys had been previously trained (see methods). As an illustration, a frame of one of these novel videos involving the baseball as the object is shown in Fig. 4a (6 to 13). Figure 4b shows the proportions of correct trials during these generalization tests for both monkeys. No bias correction was used during the entire generalization tests. Black bars indicate the performance of each monkey for the familiar action videos (90% of the trials) during the generalization tests, which is an indication of how well the monkey performed the learned classification task during that particular session. Colored bars indicate performance (proportion correct trials) for the 10% trials during which novel, untrained videos (shown in Fig. 4a) were shown (monkey M1: red bars; monkey M2: yellow bars). Asterisks indicate significant generalization (binomial test, p < 0.05). For generalization tests 1 through 6, in which a novel example of a grasping motor act was shown, significant generalization therefore indicates that monkeys categorized these novel videos correctly as ‘grasping’. For generalization tests 7 through 13 in which a novel example of a non-grasping motor act was shown, significant generalization indicates that the monkeys categorized these novel videos correctly as ‘non-grasping’. As shown in Fig. 4b, monkeys generalized to untrained grasping motor acts depicting an object grasped with a precision grip (1) or whole hand grasp (2). Both monkeys could also generalize to a novel human actor performing a grasp (3) or to a conspecific grasping (4). However, both monkeys failed to generalize to grasping performed by an artificial prosthetic arm (5). Monkeys also generalized to a novel untrained viewpoint (mirror image of the trained viewpoint). This was the case for grasping motor acts (6) as well as for the non-grasping motor acts including a hand mimicking a grasp (7) or a fist touching the surface in front of the object (8).

During training, all non-grasping motor acts involved actions directed away from the object, depicting situations during which the human hand did not touch or interact with the object. Thus a possible strategy for the monkeys to solve the categorization task might have been to categorize the videos according to objects being touched or interacted with. To investigate this possibility, we also tested the 3 familiar non-grasping motor acts (mimicked grasp, finger touch and fist touch) in a novel setting where these were directed towards the object. Videos of a hand initiating a grasp that stopped before making contact with the object were categorized correctly as a non-grasping motor act (9). While a finger touching an object was categorized correctly as a non-grasping motor act (10), both monkeys nonetheless failed to correctly categorize a closed fist touching an object and consistently categorized this motor act as a grasp (11). Note that a novel viewpoint for motor acts depicting a closed fist whereby the object was not touched (8) was correctly categorized as a non-grasping action by both subjects. Finally, we tested videos depicting two novel hand configurations interacting with the object, during which an open hand either touched the object with the palm downwards (12) or upwards (13). Interestingly, both monkeys categorized the hand palm down videos correctly as a non-grasping motor act (12), but failed to categorize the hand palm-up motor act (13). Since performance was around chance level for the latter, neither monkey confused this action with a grasp, such as they apparently did for the closed fist touching the object (11). Rather, they presumably had problems attributing this non-familiar action to either of the 2 classes, as seemed to be the case for the artificial prosthetic arm in M2 (5).

## Discussion

Action recognition is an important aspect of social cognition. Successful interaction in cooperative or competitive situations requires the ability to infer the goals and intentions of others,‘ actions. Especially since the discovery of mirror neurons^2,31^, there has been an increased interest in the neural substrates for action recognition, both in human and non-human primates.

Some of current theories as to how the brain processes information related to others’ actions^14,32–34^ are based to a large degree on single-cell evidence, describing selective neuronal responses during the observation of different actions and action settings. Although these studies have been instrumental in showing how different stimulus aspects and characteristics can influence neuronal responses, most have not directly tested action comprehension. Hence it remains difficult to conclude from such studies which aspects of the actions, such as the underlying goals and intentions of the actor or the efficacy of the action with respect the environmental constraints, a monkey comprehends when observing these actions.

Various behavioral protocols, also used in developmental and comparative research in human infants and apes, have been tested to investigate monkeys’ action comprehension abilities more directly with respect to understanding the goals and intentions of others’ actions. Some of these studies investigated monkeys’ behavioral responses while they observed rational versus irrational actions or accidental versus intentional actions^8,10,12,35^, while yet others assessed if monkeys can make a distinction between an actor that is unable versus one unwilling to perform a certain action^9,13^.

The main aim of our study was to use a two-alternative categorization task, compatible with simultaneous neurophysiological measurements, to examine if monkeys could discriminate different types of observed motor acts and were able to indicate in a straightforward manner their choice with a quantifiable behavioral response. Combining this type of operant behavioral experiments with current reversible perturbation techniques such as microstimulation, opto- and chemogenetics^36–38^, might provide additional insights into the crucial role of certain brain regions for action recognition and discrimination.

Our results show the feasibility of training monkeys to discriminate grasping motor acts from non-grasping types of manual motor acts, which are otherwise very similar. Although training took a substantial length of time (especially with monkey M1), both monkeys became very proficient in this task. Moreover, generalization tests suggest that monkeys did not merely memorize the videos or specific low-level details of the stimuli to solve the task, but could transfer the learned categorization rule to a wide range of novel untrained videos. Monkey M2, as opposed to monkey M1, had prior experience with performing a visually-guided saccade task^39^ before learning the action categorization task, which might partly explain the faster learning curve of monkey M2. However, both monkeys reached overall similar levels in performance after around 10 sessions when only a few stimuli were introduced to the stimulus batch, indicating monkey M1 did not have an overall problem learning the saccade task. While the lengthy training in our study was partly due to the setup of the experiment (introducing a large number of different stimuli to the stimuli batch over a longer period of time) and our rather strict threshold of expected performance on the task (above 80%), similar behavioral studies show that overall task performance on these tasks are both related to task difficulty and individual differences between subjects. For instance, Vangeneugden *et al*.^15^ trained monkeys on a similar task requiring their monkeys to discriminate actions depicting humanoid walkers. While the 3 monkey subjects in that study reached over 90% correct performance in as little as 10 training sessions when discrimination leftward versus rightward walking, individual differences in training length and overall performance became much more pronounced when monkeys had to discriminate forward versus backward walkers. While one of the subjects in that study reached near perfect performance on this task after 40 training sessions, their monkey M2 for instance never reached above 80% correct performance, even after 60 sessions.

Our behavioral study resulted in several interesting findings. Our data suggest that our monkeys did not interpret novel videos showing a mechanical grasping effector (prosthetic hand) in a manner similar to novel human or conspecific effectors performing the same grasp. While our experiment was not designed to test goal understanding per se, our finding seems in line with ethological observations made in rhesus monkeys and developmental studies in infants. Wood *et al*.^35^ used a two-option social foraging context and examined whether macaques would comprehend tool-related (non-biological) actions as goal-directed. While monkeys were able to interpret human actions that belonged to their own motor repertoire as goal-directed, they failed to do so when observing actions done with a pole or machete. Likewise, developmental studies suggest that 6-month-old infants respond differently to grasping actions performed by a human or an artificial effector, suggesting that infants considered only human actions as goal-directed at that early age^40^.

Although monkeys failed to generalize to an artificial grasp, one cannot conclude that monkeys fail to acquire a concept of the functionality of artificial effectors or tools, but prior observational^41^ or motor training^42^ seems to be a requirement for achieving such generalization. Previously, we have shown that rhesus monkeys can learn about the functionality of tools and are able to acquire the highly skilled motor control necessary to operate these devices^43^. Our supplementary footage (Supplementary Video S6) shows that monkeys not only can use pliers to retrieve food in a controlled way, but they also understand the functionality of certain tool features, as evidenced by their ability to correctly rotate the tool to retrieve the food in an efficient manner. Future experiments will be needed to investigate the degree to which actions done with tools or non-biological effectors generalize to novel untrained non-biological actions, after monkeys have acquired experience, either through association learning (as in our categorization task) or through physical motor experience with these tools^43,44^.

While our experiment required monkeys to discriminate ‘grasping’ from ‘non-grasping’ videos, it is difficult to assess with certainty what visual aspects of the videos the monkeys used to solve the categorization task. Our generalization data suggest that monkeys did not solve the task by merely memorizing, nor by discriminating between motor acts that involve an effector interacting with an object versus effector movements that were directed away from the object. A finger or an open hand palm touching an object were correctly categorized as non-grasping, while a non-biological effector (prosthetic arm) interacting with an object failed to be categorized correctly. The fact that a closed fist touching an object was consistently discriminated as a grasp in both animals, suggests that particular combinations of local features, for example a thumb and additional fingers in close proximity to the object may have been a particularly informative cue with which to solve the categorization task.

Since monkeys were required to fixate the entire duration of the videos before making a saccade to a target, we cannot know during which phase of the observed actions monkeys made their decision. In order to investigate this, the task could be altered by showing the 2 peripheral targets together with the action video and by allowing the monkey to make a choice at any stage of the video presentation. Our current task also required monkeys to make a saccade to the same target location for a specific action class. In order to avoid a motor bias, which doesn’t affect the current behavioral data but which could be detrimental during electrophysiology or functional imaging experiments, the task could be altered by associating each action class with a target cue differing in shape or color. After video presentation, these targets could then be displayed randomly in a balanced fashion at different locations. As an alternative, instead of using saccades as a behavioral response measurement, monkeys could also be trained to use their hands to indicate their decision, either pressing down on a button or lifting their hand^39^. When combining this categorization task with a measurement of functional brain activity to gain insights in the neural correlates of action recognition, baseline trials could be added during which monkeys fixate only a central fixation point and afterwards make their motor response (saccade or hand movement) to select either of the 2 peripheral targets to receive their reward. In addition, in order to understand the specificity of potential causal effects of focal perturbations during these type of action discrimination tasks, it will be useful also to contrast these type of tasks with similar categorization tasks requiring judgements on non-action stimuli, or action versus non-action stimuli. Finally, we should mention that we only tested a limited number of generalization settings. Related to recent observations in humans^18,45^, it may be interesting to investigate in future experiments if discrimination of observed actions in monkeys is either dependent or independent of viewpoint.

Unfamiliarity with the observed motor acts seems to be an important factor for failure to generalize to novel action displays in our experiment. Both monkeys had problems interpreting the prosthetic arm grasping and the inverted hand touching an object. While it is difficult to disentangle whether this unfamiliarity primarily reflects perceptual or motor unfamiliarity (in the case of the inverted hand), a behavioral study that investigated monkeys’ ability to evaluate the effectiveness of goal-directed motor acts^10^ showed that monkeys detect the efficacy of the goals of observed motor acts, provided they belong to the observer’s motor repertoire. In this study, monkeys’ capacity for understanding the efficacy of goal-directed and familiar motor acts failed to generalize to unfamiliar motor acts. On the other hand, other behavioral research in free-ranging rhesus monkeys suggests when assessing action outcomes, monkeys do not need to have exact motor representations of these actions^46^. With respect to visual familiarity, it is of interest that infant research shows that while 9-month-old infants do not interpret actions with a mechanical claw as goal-directed, perceptual exposure to a human operating that device, allowed 9-month old infants to interpret these actions as goal-directed^47^.

Our inverted-hand motor act that appeared in the generalization test (13) resembles a condition used in several behavioral studies investigating monkeys’ ability to differentiate intentional from accidental actions^8,12,35^. For instance, Wood and co-authors^8,35^ used a visually similar action (hand flop) and reported that monkeys (tamarins and rhesus monkeys) as well as apes (chimpanzees), selected containers potentially containing food more often when a human experimenter would touch it with an intentional motor act (hand grasp), as compared to an accidental condition, in which the human experimenter dropped his hand in a reversed manner (palm facing upwards) onto the container. The authors suggest that because this motor act is not part of the monkeys natural motor repertoire, this may have led to an inability to comprehend this gesture as a goal-directed action^35^. Although they would be physically capable of producing this type of motor behavior, they presumably have no actual motor experience with it. Using similar actions, including an inverted-hand flop as Wood and co-authors^35^, Costes-Thiré and co-workers^12^, on the other hand, failed to find evidence for discrimination between accidental and intentional actions in Tonkean macaques and capuchin monkeys. While investigating intention recognition was beyond the scope of our current experiments, similar categorization tests such as the one described here might be informative for investigating whether monkeys can indeed discriminate accidental from intentional actions.

## Conclusions

Our study shows the feasibility of training monkeys on an active action observation task in which monkeys not only had to observe actions, but had to discriminate the observed motor acts and indicate their choices with a straightforward behavioral measure. Generalization testing showed a high degree of transfer to untrained novel action videos. These types of tasks, in combination with neurophysiological recordings and reversible perturbations, will be instrumental in gaining a more in-depth understanding of the neural correlates of action recognition.

## Electronic supplementary material

Supplementary Information

Supplementary Video S1

Supplementary Video S2

Supplementary Video S3

Supplementary Video S4

Supplementary Video S5

Supplementary Video S6
