# Supplementary material for: Action Categorization in Rhesus Monkeys: discrimination of grasping from non-grasping manual motor acts
Source: Sci Rep. 2017 Nov 8;7:15094. doi: 10.1038/s41598-017-15378-6 (PMC5678109; doi:10.1038/s41598-017-15378-6)
Supplement: Supplementary file 1 — Supplementary Information [file 41598_2017_15378_MOESM1_ESM.pdf]

Action Categorization in Rhesus Monkeys: discrimination of grasping from non-grasping manual motor acts.

Koen Nelissen[1,*] and Wim Vanduffel[1,2]

[1]Laboratory for Neuro- & Psychophysiology, Department of Neurosciences, KU Leuven, Leuven, 3000, Belgium.

[2]Massachusetts General Hospital, Harvard Medical School, Athinoula A. Martino's Center for Biomedical Imaging, Charlestown, Massachusetts, 02129, USA.

[*]corresponding author:

Koen Nelissen

Laboratory for Neuro- & Psychophysiology

Department of Neurosciences, KU Leuven

Herestraat 49, 3000 Leuven, Belgium.

Email: koen.nelissen@kuleuven.be

Phone: ++32-016-330208

Supplementary material

Supplementary Video S1: Grasping motor act.

Supplementary Video S2: Finger touch front motor act.

Supplementary Video S3: Mimicked grasp motor act.

Supplementary Video S4: Fist touch motor act.

Supplementary Video S5: Finger touch side motor act.

Supplementary Video S6: Monkey using pliers.
